# Social media analysis in the context of social responsibility approach of football fan groups

**DOI:** 10.3389/fpsyg.2025.1521688

**Published:** 2025-04-22

**Authors:** Tayfun Şirin, Fahri Eryilmaz, Yeliz Eratli Şirin, Özge Aydin, Mehmet Metin

**Affiliations:** ^1^Department of Coaching Education, Faculty of Sports Sciences, Kahramanmaraş Sütçü İmam University, Maraş, Türkiye; ^2^Department of Administration and Organization, Vocational School, İstanbul Rumeli University, İstanbul, Türkiye; ^3^Department of Sports Management, Faculty of Sports Sciences, Çukurova University, Adana, Türkiye; ^4^Faculty of Sports Science, Institute of Medical Sciences, Çukurova University, Adana, Türkiye

**Keywords:** social responsibility, social media, inclusion, leadership, creativity and innovation

## Abstract

As one of sectors that address social problems, sports can pioneer very useful social responsibility efforts with its wide area of influence. Participation in social responsibility projects, especially through popularity of football, reaches much wider audience. It is seen that unofficial fan groups, which are already part of society, can direct very different sports stakeholders with their leadership positions that will affect large masses. Although it is important for this leadership to be done with active participation, it is also fact that it can be much more effective with creative and innovative projects. By examining initiatives in which in general football fans are involved within framework of social responsibility as stakeholders of football, it can be understood which problems in social area are more important from perspective of football fans. Thus, in future, much higher level of benefit can be provided for social problems by using such resources much more effectively and efficiently. In this context, this study was conducted with aim of examining social responsibility-focused Instagram posts of fan groups of three most important sports clubs operating in sports in Turkey in context of participation, leadership, innovation and creativity. In line with purpose of the study, Instagram pages, which run by fan groups, examined belong to Beşiktaş club’s Çarşı and UNIBJK fan groups, Fenerbahçe club’s Genç Fenerbahçeliler and UNIGFB fan groups, and Galatasaray club’s ultrAslan and UAUNI fan groups. 150 Instagram posts from each fan group, dating back to April 12, 2021, and total of 900 posts, were examined through content analysis. In content analysis, social responsibility-focused posts were coded and evaluated. In general, it was observed that the posts were made on axis of Agenda-Setting Approach. It is seen that social responsibility is increasingly becoming strategic necessity among sports stakeholders and is also used as means of strengthening legitimacy. In addition to leadership provided in terms of participation, it has been determined that there are areas that need to be improved in terms of creativity and innovation.

## 1 Introduction

The concept of social responsibility is related to the integration of efforts to achieve commercial and financial gain with efforts to increase the value and well-being of society in general. To ensure integration, subjective and vague concepts such as goodness, value, and hope are used (Gould et al., 2020). Yurdakul (2012) used the expression regarding the concept of social responsibility as the efforts of organizations to position themselves at a certain point and to market themselves from that point by making strategic plans for a certain problem or a certain social goal, individually or collectively or with non-governmental organizations focused on a certain area. Expressing that an organization should first ensure its financial interests when it comes to social responsibility projects, Ferauge (2012) stated that social and environmental investments should then be made, and only in this case can long-term social responsibility projects be carried out.

Football is a powerful social phenomenon that brings millions of people together and creates a collective identity (Giulianotti and Robertson, 2009). In this context, football fan groups are not only masses that support their teams but also attract attention with their sensitivity to social events and participation in social responsibility projects (Doidge, 2015). This article examines how football fans’ social responsibility behaviors are shaped, which areas they focus on, and why certain issues are given more importance from the perspective of the agenda-setting approach. Agenda-setting theory argues that media and social groups influence which issues are highlighted on the social agenda (McCombs and Shaw, 1972). Fan groups can be effective in bringing certain issues to the forefront both in their own communities and in society in general through social media (Sanderson, 2014). Fan organizations, having a high number of members, people from different socio-economic backgrounds coming together, loyalty to the same sports club and CSR projects both represent the football club they are affiliated with and reflect the orientation of football clubs in society. Social media platforms have a significant impact on communication in the field of sports thanks to the interactive features they offer; they have quickly gained acceptance for both fans and athletes (Kassing and Sanderson, 2010). Social media is a valuable tool for CSR because it can help build a charitable brand/image and provide stakeholders with immediate/up-to-date information (Formentin and Babiak, 2014). Some scholars (Ali et al., 2015) argue that using social media to communicate social responsibility activities is an effective way to establish good relationships with different stakeholders. Most importantly, teams and professional athletes with millions of followers on social media platforms can mobilize their followers to act and participate in helping others (Bennett, 2014). In this context, this study aims to investigate the aims of fan groups in CSR activities through social media; It is aimed to reveal the current situation and deficiencies in contributing to social benefit and spreading social awareness, receiving requests for CSR activities from those in need, establishing partnerships with civil society organizations, collecting donations, and creating income items. When the literature is reviewed, it is considered that there are very few studies that present a situation or analysis regarding the CSR activities of football club fans, so this research focuses on the CSR activities of fan organizations and evaluates the aims and areas of CSR activities. In this context, when the posts made by football fan groups on social media are examined in this research, it will be determined which topics are the most shared social responsibility areas, which topics are the least shared one. For example, social responsibility projects based on condolence and commemoration have a content that unifies and emphasizes the spirit of solidarity for football fans. Commemoration events held in cases of the loss of important football players, club legends or fans attract great attention on social media. This situation shows the importance that football communities attach to the process of creating collective memory and identity (Brown, 2017). In contrast, topics such as “poverty” and “education” are less on the social media agenda of fan groups. One of the reasons for this is that these topics usually require long-term solutions (Bale, 2000). Since the actions of fan groups are usually spontaneous and based on emotional foundations, events that require immediate reactions (such as condolences and natural disaster aid) attract more attention (Pope and Williams, 2011). Issues such as poverty and education are less embraced by fan groups because they require longer-term planning and resources.

Considering that CSR research in sports is still in its infancy, we have kept the scope of our study within certain limits. First, our article addresses CSR in professional sports. In particular, the study focuses on the role played by social media in communicating socially responsible activities carried out by professional team fan groups. The emotional bond and passion that fans establish with their football team is also very important in terms of the awareness of football clubs’ CSR messages in society (Babiak and Wolfe, 2009). In addition, the fact that football’s place in popular culture is built more on “instant emotional reactions and victories” can reduce interest in long-term social responsibility projects (Walters and Chadwick, 2009). Fan groups focus more on issues that have an immediate impact on social media platforms and spread quickly within the community (Rowe, 2013). From the perspective of agenda-setting theory, the prominence of issues such as “condolence/commemoration” in the social media posts of football fan groups shows the importance these groups give to collective identity, solidarity and emotional sharing. In contrast, issues that require long-term solutions such as “poverty” and “education” receive less attention. This study uses agenda-setting theory to understand the nature of football fans’ social responsibility behaviors and reveals how social media and fan participation are shaped.

## 2 Literature review

### 2.1 Leadership and social responsibility

Strand (2011)'s approach to the subject of social responsibility and leadership can be summarized as follows: How consistently can it be argued that CEOs or business people, who have the leeway that the economic and legal structure allows to earn more money, disregard all kinds of scandals, bailouts, financial crises, on the one hand, while on the other hand, they embrace the values of social responsibility. Based on this, the effort to integrate an ambiguous subject such as social responsibility within the complex structure of leadership is difficult both because of the breadth of concepts and the contradictory relationship between concepts. Silvestri and Veltri (2020) touched upon two issues in examining the relationship between leadership and social responsibility. The first of these is the individual approach of the leader and what the leader brings to the organizational approach of social responsibility; the second is the examination of the results of the leader’s influence on the social responsibility process. Leaders who understand that organizations, whether they are for profit or not, are a part of society should act on the assumption that social problems can be an obstacle for themselves and their institutions and should focus on the right social responsibility area. In doing so, the most important weapon of leaders is to take responsibility in such projects and to exhibit behaviors that will encourage their followers to take responsibility. The way should be paved for leaders who can apply types of leadership such as servant leadership and ethical leadership (Gorski, 2017). Trait and behavioral leadership theories, while addressing the projects and outcomes of leaders in the field of social responsibility, focus on the personal differences of leaders or the behavioral differences of leaders, respectively. In addition, while trait theories see social responsibility as something left in the hands of the leader, behavioral theories code social responsibility as an area that can be learned, trained about, and applied. In addition, it is obvious that ethical, responsible, and servant leadership approaches among modern leadership theories are more prone to social responsibility because they attach less importance to it in financial terms (Silvestri and Veltri, 2020). In this context, Tourigny et al. (2019), who examined the effect of ethical leadership on social responsibility, which can be put forward as an example, revealed that ethical leadership is important in creating awareness about social responsibility and shaping the perception of social responsibility, that the common awareness created has an important role in reinforcing institutional trust, and that with increasing institutional trust, employees’ tendency to take on additional responsibilities increases. In another study, it was found that ethical leadership practices increased organizational performance in operational, commercial, and economic terms through social responsibility projects. On the other hand, to stand out, make a difference, and even increase profit margins compared to other competitors in the sector, it is necessary to pay special attention to the philanthropic activities of social responsibility and the levels of these activities (Kim and Thapa, 2018). As another example, it has been shown that the financial position of organizations is the main obstacle to the adoption of social responsibility projects; internal and external stakeholders and ethical leadership reinforced with personal values are very important success factors for social responsibility (Saha et al., 2020). As a different leadership theory, it has been observed that the added value of social responsibility projects increases with stakeholder-focused marketing integrated with transformational leadership. All things being equal, transactional leadership has produced a more positive effect on social responsibility practices and organizational outcomes compared to transformational leadership. In this context, it can be said that transformational leadership requires mediating variables such as stakeholder-oriented marketing. This shows that there is a complementary situation between leadership and marketing in terms of social responsibility projects (Du et al., 2013). Khan et al. (2018), who found that social responsibility practices as well as innovation and performance were positively affected in organizations managed with a transformational leadership style, found that traditional leadership styles were behind in this regard.

### 2.2 Inclusion and social responsibility

A study conducted by Hon and Gamor (2022) found that inclusion in social responsibility projects can create a positive perception of the reputation of organizations from the perspective of stakeholders. In another study, it was observed that organizations experiencing reputation loss increased their official social responsibility inclusion, and it was determined that they both expanded their social responsibility portfolios and adopted different social responsibility policies (Jung et al., 2024). With social responsibility inclusion, many large and small organizations will be able to address strategic concerns about a sustainable world, employee and customer satisfaction and well-being, and fair trade. This shows that with inclusion in social responsibility, corporate policies and practices focused on stakeholders, performance or motivation, and obligations to those directly and indirectly affected by this can be fulfilled through a more inclusive approach (Hon and Gamor, 2022). Nijhof et al. (2002), who presented a model for organizations to integrate social responsibility within themselves, mentioned that the model has four different processes. These can be listed as the consultation process required to draw a common path with stakeholders; the integration process that ensures identification with the institution after determining the right project; the information process where the actions taken within the subject are shared with stakeholders; and the evaluation process where each stakeholder makes their own positive or negative assessments on the subject and provides feedback. With the correct management of this process, social responsibility activities will be able to act on much more solid foundations. As a different example, Afzal et al. (2023), who examined the period between 2011 and 2018, stated that social responsibility expenditures in the banking sector have shown a continuous increase, but financial inclusion in this context has not led to a change in the demand for banks’ products. They stated that these social responsibility expenditures are distributed in seven categories: education, health and security, humanitarian and disaster relief, arts and culture, environment, infrastructure, and others.

### 2.3 Creativity and social responsibility

Creativity is the starting point of innovation and refers to a mental development process that will create a combination of new ideas and inventions and existing ideas and inventions that will penetrate every aspect of the work to be done (Yurdakul, 2012). In works done using creativity, individuals rarely state that they have done a process, or a production based on scientifically explained creativity theories. Theories that will explain the behaviors of the person who realizes the phenomenon called creativity to make daily life beautiful rarely or never touch on this subject (Lim and Plucker, 2001). Although the brainstorming method was used usefully for a while in terms of diversifying social responsibility projects, it has become clear that events and facts need to be approached with a wider imagination to evaluate much wider opportunities and possibilities (Yurdakul, 2012). In the study conducted by Chaudhary and Akhouri (2018), it was stated that the perceptions of people within an organization toward the social responsibility projects carried out by the organization are not always positive. It has been observed that these projects attributed to internal reasons provide more positive results in terms of inclusion and creativity; whereas projects attributed to external reasons, i.e., organizational interests, do not produce an effect in terms of inclusion and creativity.

### 2.4 Innovation and social responsibility

Kim et al. (2014), concluded that social responsibility projects involving innovation are carried out for organizations in the short term and focused on interest on the one hand, and long term and focused on R&D and discovery on the other, suggesting that innovation experiments carried out within social responsibility should be selected correctly by the leaders within the organization according to the focus of the investment type. Leaders within the organization must not lose their focus to use the innovation opportunities that come their way. When they direct this focus to social responsibility, they can provide the emergence of previously unthinkable beneficial results with innovation opportunities added to the concepts such as moral obligation, sustainability, and respect attributed to the importance of social responsibility. However, to ensure this, the organizational culture must also have a structure that allows this (Asongu, 2007). Crets and Celer (2013), who stated that organizations that create long-term value by using innovation strategically in terms of social responsibility will catch important growth opportunities, drew attention to three elements. These are, respectively, a highly developed social responsibility management that embraces transparency, the adoption of social innovation as a business strategy, and cooperation with stakeholders. The authors argue that organizations that adopt these elements can demonstrate leadership by approaching ethical values, people, communities, and the environment with respect while going beyond the relevant legislation.

In the study conducted by Amos (2017), the connection between the leadership of institutions and the innovative perspective of individual employees of these leaders was mentioned among the elements that could affect social responsibility activities, and it was stated that with such a development, both the competitive power would increase and coordination between the economic value to be gained and the social value could be provided. It is stated that while bringing a different perspective to social responsibility projects with innovation, it is necessary to focus on large-scale systematic changes as well as quality, effectiveness, and sustainability. In doing so, it is necessary to first connect social responsibility to the basic activities and expertise of the institution within the organizational structure with innovation. Then, opportunities such as project production, permission, and assignment can be created to encourage human resources for voluntary work. In addition to these; preparing and providing an area that will encourage employees to take such initiatives based on their in-house roles, developing some in-service training in this context, highlighting leaders who attach importance to ethics related to social responsibility within the institution, and creating a scheme that will ensure that the initiatives to be carried out by the organization become part of a longer-term plan can be listed as other steps (Googins, 2013). Asongu (2007) touched upon five issues regarding the use of social responsibility innovations for organizational advantage. These include turning environmental groups into stakeholders, making environmental commitments a part of organizational policy, correctly predicting social needs and intervening before they become a problem, producing all kinds of products of the organization in a clean manner that will keep them away from a bad image, and finding a place to move ahead of the social responsibility curve with rules and regulations to be developed within the organization (Asongu, 2007).

### 2.5 Sports, football and fan groups and social responsibility

In general, sports develop social responsibility projects in terms of scope and quality with some elements it includes such as communication, media, health, youth, socialization, and sustainability (Smith and Westerbeek, 2007). Sports businesses that have adopted the concept of social responsibility and allocated research resources expand and increase the impact of social responsibility projects by using their recognition in the media, the way they are perceived by society, the awareness of their employees, especially athletes, and their social interaction power. Social responsibility projects, whose impact increases and expands, are also becoming more important for sports (Sönmezoğlu et al., 2013). While the applications that meet on the common ground of sports and social responsibility are increasing, the social responsibility applications within the scientific product and sports industry are also expanding (Babiak and Wolfe, 2006; Smith and Westerbeek, 2007; Breitbarth and Harris, 2008; Babiak and Wolfe, 2009; Walters, 2009; Sheth and Babiak, 2010). It has been determined that professional sports clubs, athletes, and philanthropic organizations in the USA contribute approximately 100 million dollars annually to society while directing their financial power to social responsibility projects aimed at society (Sheth and Babiak, 2010). In this sense, it can be said that the importance of the social roles of sports organizations that create strong financial structures has increased (Hamil and Morrow, 2011). Therefore, it can be stated that sports are now an important tool in the spread and development of social responsibility. Sports-related organizations have gained a great opportunity in terms of contributing to society by using this tool (Smith and Westerbeek, 2007). In the process of evaluating this opportunity, it was observed that the institutionalization moves made in professional sports regarding Corporate Social Responsibility (CSR) were under the influence and pressure of the internal and external stakeholders of the sport (Babiak and Wolfe, 2009). The connection between football and social responsibility can be explained through England, which is considered the cradle of football. England, which experienced difficulties in football environments in the 1970s and 1980s, prepared and implemented a roadmap that would strengthen it first in terms of legal and administrative aspects and then financially with the changes it initiated at the end of the 1980s. In this process, the ownership of the clubs and the damage to the fan ties caused the clubs to turn to social responsibility projects to strengthen their relationship with society and the fans, and the government, relevant federations, clubs, and civil society organizations had significant effects on this tendency (Brown et al., 2006).

### 2.6 Social media and social responsibility

The fact that the increase in social responsibility projects, especially after 2007 (Walters, 2009), coincides with the birth of social media is also important in terms of sports, marketing, and public relations. Howard and Parks (2011) state that social media are platforms that offer a wide variety of social content, beyond being just a personal tool. This situation causes the phenomenon of sports, which have an inherent interaction, to be transferred to these platforms. However, this transformation process experienced with social media also encompasses the fans and viewers, who are the target audience of a large sector such as the sports industry. As an economic, cultural, and political phenomenon, sport is becoming an extremely important area of consumption for the media (Demir and Talimciler, 2015). Mass distribution of news about sports events has begun to be done effectively through network-based social media tools as well as traditional communication tools. Sports fans/viewers, who are in a close relationship with information and communication technologies, have started to follow the statements of managers, athletes, and coaches through these new media tools that are easy to use and fast, in addition to reaching all kinds of results regarding the competitions. Especially the two-way communication process that social networks have brought about, and the fact that sports are more visible in the media have also started to be effective in terms of fans and viewers supporting teams from other countries (Demir and Talimciler, 2015). In the early 21st century, when new media tools started to become popular, it was seen that many innovations emerged in the sports industry. These innovations that emerged depending on the developments in the technological field have of course made the necessity and use of these social networks mandatory in sports institutions. In parallel with these developments, the fact that internet use is determined as 90% among fan/viewer groups, which are predominantly young (Demir and Talimciler, 2015), has highlighted the necessity of using new media in terms of the target audience that sports organizations serve. This necessity has also been reflected in the research. Three concepts are highlighted in the theoretical model created based on NFL fans’ social media use; access, voice, and validation. When we look at these concepts; the concept of access with listening, researching, and gathering information, accessibility to players and teams, establishing parasocial ties; the concept of voice with legitimizing fandom in the eyes of the society, communicating with other fans in terms of socialization, creating alternative identities; the concept of validation that meets the need for the fan’s voice to be listened to, rather than the voice of the athletes and the sports media, and for this to be answered by someone else, especially the athletes and the sports media (Martin, 2012). Based on this model, it can be stated that the boundaries of the interaction between sports clubs, spectators, and athletes have expanded. In addition, when it is considered that the social responsibility projects carried out will contribute positively to both the local and global images of sports clubs; in the case of football, the most dynamic area where the football-social media connection can increase the image contribution in question together with social responsibility is the fan communities.

## 3 Purpose of the study

The purpose of this study, which was conducted to address the concepts of leadership, inclusion, creativity, innovation, sports, football, fan groups, and social media, the relationship between which is explained one by one above with social responsibility, is to examine the social responsibility-focused Instagram posts of the fan groups of the three most important sports clubs operating in Turkey on social media platforms in the context of inclusion, leadership, innovation, and creativity. In addition to the literature given above, the suggestion of Chaudhary and Akhouri (2018) examines the connection between social responsibility and creativity as well as the research on inclusion, and Ferauge (2012) touched on innovation and social responsibility, and the suggestion that different subject headings could be integrated next to these two headings came to the fore. Adding that innovation seems to be an important tool in integrating social responsibility into organizational culture and policy, Ferauge (2012) predicts that organizations will start to focus on environmental and social issues with innovation after they increase their economic earnings to a certain level. However, he stated that for this to become permanent, innovation must be made sustainable and that the most important factor that will ensure this permanence is leadership. In the study conducted by Saha et al. (2020), they mentioned that the studies conducted on leadership and social responsibility issues are mostly related to the production sector and underlined that these studies should also be conducted for different sectors. In this study, football, which is evaluated within the entertainment sector, and even fan groups, which are both stakeholders and customers of this entertainment sector, are taken into consideration. In the process where fans first become followers and then representatives of the club with social media, the content of being a fan has also expanded. For this reason, the fact that fan groups are active in social responsibility provides an opportunity for both the club, the fan community and the direct fans to be seen as sympathetic and for the club’s name to be mentioned with positive events. In this context, the necessity of conducting research that will analyze the content of social media used to announce social responsibility projects has emerged, and this research aims to fulfill this requirement. The study examining the social media posts of the fan groups of Beşiktaş, Fenerbahçe, and Galatasaray clubs in Turkey (Olca, 2020) has taken a step toward understanding and explaining the perspective of Turkish clubs and Turkish fans on social media. Finally, as Yurdakul (2012) stated; the principle that combining two unrelated ideas is the strongest among creativity methods has also constituted one of the creative inspirations for this study, and a study was designed focusing on the social responsibility projects in which football fan groups participated and the leadership, creativity and innovations in the field of social responsibility they demonstrated through social media. This study will take another important step by examining the social media posts of fan groups in terms of social responsibility.

## 4 Methods

In this study, which was conducted with the aim of examining the social responsibility-focused Instagram posts of the fan groups of the three most important sports clubs operating in Turkey in the context of inclusion, leadership, innovation and creativity, the largest and most effective fan groups of Beşiktaş, Fenerbahçe and Galatasaray, which are the clubs operating in Turkey and have the highest number of fans in football, the most popular branch in the country, were taken as a sample. In Turkey, fan groups are actively working on disaster relief, environmental awareness and education projects, parallel to similar fan movements in Europe. For example, during the February 6, 2023, Kahraman Maraş Earthquake, fans of the three major clubs organized a serious effort via social media to collect, distribute and deliver humanitarian aid to those in need. Their rapid organization, especially in disaster relief, can be shown as a stronger example of solidarity than the examples in Europe. These fan groups were determined as Çarşı, Genç Fenerbahçeliler (GFB) and ultrAslan (UA), respectively, and university fan groups formed by university students were also examined within these fan groups. These fan groups were determined as UNIBJK, UNIGFB and ULTRASLANUNI.

The Instagram accounts of the six fan groups have been retrospectively reviewed as of April 12, 2021. It was analyzed using content analysis to include 900 posts in total, 150 from each fan group. The date of first post, total number of posts and number of followers were recorded on specified date. The reason for choosing Instagram accounts is that number of followers of this social media platform in Turkey is higher than others. The data that is subject of research are posts related to social responsibility in 150 posts obtained on Instagram accounts of fan groups. A codebook was created to be used in analysis of posts. While creating codebook, conceptual framework and research conducted within framework of social responsibility theory related to research topic were used (Olca, 2020; Grover et al., 2019). While this coding is done, it is aimed to include topics in social responsibility theory to determine social responsibility tendencies of fan groups. In coding process for this purpose, firstly, posts were classified according to their categories. Categories were determined based on the classifications made on the basis of the studies conducted by Olca and Grover et al. In this context, before starting the research, the sharing contents of the fan groups in different periods were examined and some categories that could give the researchers an idea within the scope of philanthropy were determined based on the studies of Olca and Grover et al. The titles of these categories are: Social message, Advertising, Campaign, Announcement, Celebration, Condolences/ Commemoration, Gender Equality (Gender), Poverty, Environmental Awareness, Education, Inequality-Discrimination.

## 5 Findings

Table 1 presents the total number of followers, total number of posts and first post date of the six fan groups whose Instagram accounts were examined. The UltrAslan fan group has the highest total number of followers and is also notable as the fan group that has been using Instagram for the longest time among the six fan groups. In contrast, Çarşı, which has almost a quarter of the number of followers of UA, was found to be the most active fan group using Instagram. It was determined that the university structures of the fan groups were more passive on Instagram than the fan group itself, i.e., had fewer posts. It was observed that the university structures of the fan groups, except for UA, started using social media before the fan group itself.

**Table 1:** Numerical data of fan groups Instagram accounts.

Fan group	Total number of followers	Total number of Instagram sports	First Instagram posts
ÇARŞI	523.000	13.577	20.07.2014
UNIBJK	11.200	1.802	25.02.2014
GFB	526.000	2.382	01.05.2018
UNIGFB	59.600	1.774	16.09.2015
UA	2.200.000	7.933	30.03.2013
UAUNI	530.000	4.234	30.01.2014

Table 2 shows the findings obtained from the content analysis of Instagram posts made by fan groups within the scope of social responsibility.

**Table 2:** Distribution of Instagram posts of fan groups related to social responsibility by fan groups and topics.

	ÇARŞI		UNIBJK		GFB		UNIGFB		UA		UAUNI		Total	
	*N*	%	*N*	%	*N*	%	*N*	%	*N*	%	*N*	%	*N*	%
Social message	2	1.33	2	1.33	1	0.67	7	4.67	8	5.33	5	3.33	25	2.78
Advertising	3	2.00	0	0	0	0	0	0	0	0	0	0	3	0.33
Campaign	6	4.00	3	2.00	1	0.67	1	0.67	1	0.67	1	0.67	13	1.44
Announcement	1	0.67	4	2.67	1	0.67	0	0	0	0	0	0	6	0.67
Celebration	1	0.67	9	6.00	6	4.00	8	5.33	7	4.67	1	0.67	32	3.56
Condolences/commemoration	3	2.00	22	14.67	6	4.00	7	4.67	11	7.33	6	4.00	55	6.11
Gender equality (Gender)	0	0	4	2.67	0	0	3	2.00	1	0.67	3	2.00	11	1.22
Poverty	0	0	0	0	0	0	0	0	0	0	0	0	0	0
Education	1	0.67	0	0	0	0	0	0	0	0	0	0	1	0.11
Inequality/discrimination	0	0	1	0.67	0	0	0	0	0	0	0	0	1	0.11
Environmental awareness	0	0	1	0.67	0	0	0	0	0	0	0	0	1	0.11
SR sum	17	11.33	46	30.67	15	10.00	26	17.33	28	18.67	16	10.67	148	16.44
Posts in other areas	133	88.67	104	69.33	135	90.00	124	82.67	122	81.33	134	89.33	752	83.56
Sum	150	100	150	100	150	100	150	100	150	100	150	100	900	100

It was determined that 148 (16.44%) of the 900 posts examined were related to social responsibility. It was observed that 83.56% of the examined posts were about topics such as transfers and match results that were not related to social responsibility. The posts were listed from the most shared to the least shared as condolence/commemoration (6.11%), celebration (3.56%), social message (2.78%), campaign (1.44%), gender equality/gender (1.22%), announcement (0.67%), advertisement (0.33%), and at the bottom of the list were education, inequalities/discrimination, and environmental awareness, which were shared only 1 (0.11%) time. It was observed that no posts were made about poverty.

According to the findings, the number of posts about social responsibility among the fan groups is listed from most to least as UNIBJK (%30.67), UA (18.67), UNIGFB (%17.33), Çarşı (11.33), UAUNI (10.67), GFB (10.00).

When the posts made according to social responsibility topics are examined one by one in the fan groups, the findings are presented below:

- It was determined that there were 17 (%11.33) posts about social responsibility among the 150 posts examined by the Çarşı fan group. It was determined that the topics of these posts were listed from most to least as campaign (%4.0), condolence/commemoration (%2.0), advertisement (%2.0), social message (%1.33), announcement (%0.67), celebration (%0.67), education (%0.67). It was found that no posts were shared on gender equality/social gender, poverty, inequalities/discrimination, environmental awareness.
- It was determined that there were 46 posts (30.67%) about social responsibility among the 150 posts examined by the UNIBJK fan group. It was determined that the topics of these posts were listed from most to least as condolence/commemoration (14.67%), celebration (6.0%), announcement (2.67%), gender equality/social gender (2.67%), campaign (2.0%), social message (1.33%), inequalities/discrimination (0.67%), environmental awareness (0.67%). It was found that no posts were shared about advertising, poverty, or education. It was determined that there were 15 posts (10.0%) about social responsibility among the 150 posts examined by the GFB fan group. It was determined that the topics of these posts were listed from most to least as celebration (4.0%), condolence/commemoration (4.0%), campaign (0.67%), social message (0.67%), announcement (0.67%). It was found that no posts were shared on the subjects of gender equality/social gender, poverty, inequalities/discrimination, environmental awareness, advertising, and education.
- It was found that 26 posts (17.33%) were about social responsibility among the 150 posts examined by the UNIGFB fan group. It was found that the subjects of these posts were listed from most to least as celebration (5.33%), condolence/commemoration (4.67%), social message (4.67%), gender equality/social gender (2.0%), and campaign (0.67%). It was found that no posts were shared on the subjects of announcements, poverty, advertising, inequalities/discrimination, environmental awareness, and education.
- It was found that 28 posts (18.67%) were about social responsibility among the 150 posts examined by the UA fan group. It was determined that the topics of these posts were listed from most to least as condolence/commemoration (%7.33), social message (%5.33), celebration (%4.67), campaign (%0.67), gender equality/gender (%0.67). It was found that no posts were shared on the subjects of poverty, inequalities/discrimination, advertisement, announcement, environmental awareness, or education. • It was determined that 16 (%10.67) posts were made on social responsibility among the 150 posts examined by the UAUNI fan group. It was determined that the topics of these posts were listed from most to least as condolence/commemoration (%4.0), social message (%3.33), gender equality/gender (%2.0), celebration (%0.67), campaign (%0.67). It was found that no posts were shared on the subjects of advertisement, announcement, poverty, education, inequalities/discrimination, or environmental awareness.

## 6 Discussion

In this study, which was conducted with the aim of examining the social responsibility-focused Instagram posts of the fan groups of the three most important sports clubs operating in Turkey in the context of inclusion, leadership, innovation and creativity, the most well-known and most active fan groups of Beşiktaş, Fenerbahçe and Galatasaray, the three football teams with the most fans in Turkey, were examined. The social responsibility-focused posts made by Çarşı, Genç Fenerbahçeliler and UltrAslan and the university structures of these fan groups, UNIBJK, UNIGFB and UAUNI, on Instagram were examined.

It was observed that 16.44% of the posts examined within the scope of the study had social responsibility content. In the study conducted by López-Carril and Anagnostopoulos (2020), it was determined that only 2% of the social responsibility posts focused on Covid-19 on the Instagram platform of sports clubs in LaLiga. In addition, it was stated that with these posts, clubs could “reinforce their legitimacy by strengthening their role and reputation in society” (López-Carril and Anagnostopoulos, 2020). The most shared topic by the fan groups in our study was condolences/commemorations. It can be said that the topic of condolences/commemorations, which is one of the topics that almost every human community in the world attaches great importance to, is also expressed by fan groups on social media. This situation shows that the historical commitments of fan groups have reached a certain maturity and that they can realize this commitment not only through traditional means but also through new media. It also shows that the transformation of fan groups, which can be evaluated within the traditional concept of community (Subaşı, 2005), into virtual communities (Rheingold, 2000) has begun. In addition, another finding that shows the unity behaviors displayed by fan groups gathered around a certain identity, leaving individual differences aside, is that the second most shared topic of social responsibility is celebrations. The fact that fans who experience the act of being happy and sad together in every match carry the same feelings to their digital identities within the network society (Castells, 2008; Van Dijk, 2006) is the reason for the statement, “Football is never just football.” Kuper (2003) also shows how much is behind his words and how important a tool football is from a sociological perspective, which is also compatible with the ‘Agenda Setting Approach’ put forward by McCombs and Shaw in 1972. This is also reflected in our findings. The use of football as a tool to change the agenda also indicates certain leadership behaviors in the field of social responsibility. However, it is also seen in our findings that the topics shared are far from creativity and innovation. In this context, the fact that no posts were made about poverty, only one post was made about education, inequality/discrimination, and environmental awareness, and the fact that the posts on these three topics came from Beşiktaş fan groups show that the social problems that have come to the fore in Turkey in recent times are ignored by the fans and fan groups. The absence of a post about poverty in an environment where per capita income is decreasing and the hunger line and minimum wage go parallel; the fact that education is not discussed in an area where a basic education strategy in primary, secondary and higher education changes as governments change and where there is a constant brain drain; the fact that inequality/discrimination is not mentioned in an environment where there is a racial terror problem that has been going on for almost 40 years and immigration crises; The fact that environmental awareness is ignored in an environment where people are unprepared for natural disasters (such as forest fires, floods, epidemics) is an indication that football is a tool for manipulation and agenda changing. On the other hand, it should be noted that the Çarşı group (Dikici, 2008), which is famous for its opposition in political issues, and UNIBJK, when considered together, participate in almost every issue except poverty. The research data indicating that 79.1% of the organizations that consider both the public and the institutional benefits in social responsibility activities publish such activities on their corporate websites; on the other hand, 67.6% of the organizations that state that they carry out social responsibility only for the public benefit do not publish their social responsibility activities on their corporate websites (Sert, 2012) form the basis for the relations with the power centers we mentioned regarding fan groups, while on the other hand, it also shows that fan groups take some steps to position themselves as a power center directly. This also reveals that fan groups show leadership within the framework of social responsibility. On the other hand, it also supports the views of professional sports managers who describe social responsibility as a strategic necessity (Sheth and Babiak, 2010) and the view that sees social responsibility as a means of reinforcing legitimacy (López-Carril and Anagnostopoulos, 2020). These posts made on social media make both the fan group, the club and the stakeholders related to the club and the fan group heroes in the eyes of the society and facilitate their reaching some material and moral gains. In this context, it can be stated that the two-way club-fan interaction model stated within the scope of a study focused on branding and social media in football (Parganas et al., 2015) can be expanded by adding stakeholders and segments not related to football through social responsibility.

The fact that football fan groups focus on “condolence/commemoration” topics on social media is a phenomenon that reinforces their collective identity and emotional bonds. Major disasters, deaths of former football players or major accidents are among the events that deeply affect this collective. For this reason, the commemoration of such events receives great support from fans. At the same time, condolence and commemoration messages are usually short, easy to share and instantly interactive content. Sharing a condolence message, especially on social media platforms, requires less time and resources than actively supporting an education or poverty project. Especially in events such as mourning, which are among the important values and rituals of Turkish society and are passed down from generation to generation, society is more sensitive to emotionally intense topics such as condolences and being with the lost. It can be said that fans reinforce their sense of being a part of their team and group by providing emotional unity in the face of losses and taking on the role of a social actor. In this context, it can be said that fan groups support this process by sharing the same content, as the official social media accounts of the clubs also highlight such posts.

However, it can be predicted that the reason why they do not show enough tendency toward more sustainable projects such as “poverty” and “education” within the framework of social responsibility is because these projects require financial support. Since a large part of fan groups are organized with individual donations or on a voluntary basis, they may not have sufficient resources to allocate to such projects. At the same time, since poverty and education projects are projects that require long-term and sustainable solutions rather than providing instant interaction, it can be thought that fan groups are generally more interested in content that can be consumed quickly and easily interacted with. This reveals that awareness in these areas needs to be increased. Clubs, fan groups and civil society organizations can increase the visibility of such projects and encourage fans to engage in long-term social responsibility activities by collaborating. It should not be forgotten that football is not just a game; it can be a tool for social change. If fan groups go beyond “condolence/commemoration” posts and focus more on issues such as education, combating poverty and environmental sustainability, this will greatly contribute to increasing social benefit.

When we look at the fan groups specifically, it is seen that the UNIBJK fan group is the most active community in sharing posts about social responsibility. It can be said that these fan groups, which are university students, care more about social responsibility because they represent a segment that has reached a higher level of education and therefore has a broader perspective. In terms of clubs, it was observed that only Galatasaray’s university fan group made fewer social responsibility posts compared to the main fan group. Beşiktaş and Fenerbahçe’s university fan groups were found to make more posts. However, since the topics shared were similar within the fan groups of the clubs, it can be claimed that the fan group and the university structure are prone to similar social responsibility projects. In other words, it is very normal for the variety of social responsibility works structured in echo chambers (Jamieson and Cappella, 2008) that emerge from the filter bubbles (Pariser, 2011) created by a social group that one enters as a fan of a team to be limited. In this context, the homophily phenomenon related to public debate and idea generation over the internet can also be mentioned (Özçetin et al., 2012). In support of this, the most frequently shared social responsibility topic of all fan groups except the Çarşı group was found to be condolence/commemoration. In the Çarşı group, the most frequently shared social responsibility topic was the campaign social responsibility topic. The content of these posts includes aid campaigns for children with SMA, which have been on the agenda recently. It has been determined in this context that the Çarşı group demonstrates a very valuable leadership example in terms of social responsibility. This situation also shows that the Çarşı group’s approach to social responsibility is both to have a wide social responsibility portfolio and to act within a creative and innovative structure that will expand this portfolio. It has been determined in this context that the Çarşı group has demonstrated a very valuable leadership example in terms of social responsibility. This situation also shows that the Çarşı group’s approach to social responsibility is to have a wide social responsibility portfolio and to act within a creative and innovative structure that will expand this portfolio. In this sense, the Çarşı group encourages inclusion with its leadership in social responsibility, which it maintains with a creative and innovative understanding, and the university structure of the group has also undertaken a significant number of social responsibility projects in terms of quantity.

Finally, it is necessary to mention separately the sharing of the social responsibility process via social media. In this context, in line with Martin (2012)'s theory on social media use and fans, social responsibility-focused Instagram posts show that fans research and gather information on social issues, can establish parasocial ties, try to legitimize themselves in the eyes of society both as individuals and fans, can socialize with other fans outside of football, try to create alternative identities for themselves through fandom, see their own voice as valuable and share it with the public, and fill the points of access, voice and approval. In this context, our study is a study that supports Martin’s theory.

It has been observed that Çarşı and UNIBJK groups, which are related to Beşiktaş team, carry out more extensive social responsibility projects compared to fans of other clubs by acting with an approach that covers different issues. In addition, health-focused posts made within the scope of social responsibility also reveal different aspects of football fans who are sometimes identified with violence. When fans, who have been seen only as the cause of football violence for many years, are evaluated with different dynamics, it shows that the perception of fans in society is mainly shaped by the news presented by the mainstream media. Studies conducted with both people in the sports media and other stakeholders in sports have shown that the mainstream media reflects news focused on ratings, bias, and violence (Özsoy, 2009; İlhan and Çimen, 2011; Özsoy, 2012; Ünsal and Ramazanoğlu, 2013; Taşmektepligil et al., 2017), which also grounds the mainstream sports media in shaping public perception in terms of the ‘Agenda Setting Approach’ (McCombs and Shaw, 1972). For this reason, these posts made on social media outside the mainstream media are important both in terms of providing an opportunity to correct misperceptions about sports culture and in terms of revealing that the mainstream media does not reflect society as well as social media. However, it should be particularly noted that the issue should be evaluated specifically in the mainstream Turkish sports media. It has been observed that Turkey’s important written sports newspapers carry their news focused on football and the big three to their social media posts (Kürkçü, 2016). On the other hand, a study conducted on mainstream sports media in the United States found that issues of discrimination such as race, religion, and sexual orientation, which are frequently discussed in American society, are frequently covered in mainstream media (Schmidt, 2018). This shows that an impartial sports media can voice important issues that concern society in general. Therefore, the fact that a fan group in Turkey, which is claimed to be anarchist and dissident, cannot share social responsibility for important issues in society is important in terms of showing the pressure from the media and society in the country.

Sports clubs are seen not only as competitive organizations but also as important actors that promote social change (Smith and Westerbeek, 2007). While social responsibility (CSR) is gaining more and more importance in the world of sports, fan groups and digital platforms are at the center of this process (Breitbarth and Harris, 2008). In the digital age, fan participation plays a critical role in expanding and deepening clubs’ social responsibility activities. The sports industry is increasingly playing a role in the field of social responsibility. Breitbarth and Harris (2008) state that sports clubs consider social responsibility as an opportunity to provide social benefits both in local communities and on a global scale. In this context, it is suggested that sport is a powerful tool in raising awareness on social responsibility issues such as health, education, environmental awareness and equality (Babiak and Wolfe, 2009). However, for social responsibility initiatives to be successful, not only clubs but also fans need to take an active role in this process. Digital platforms can increase the impact of social responsibility projects by enabling fans to interact with clubs and social issues (Walters and Tacon, 2011). Fans’ social media interactions are important data that clubs can use to make their social responsibility initiatives more effective. Using this data, clubs can develop strategies such as developing content strategies by focusing on the social responsibility issues that their fans interact with the most, supporting social responsibility projects with content directly created by fans to ensure the active participation of fan groups, organizing awareness campaigns on social media platforms to raise fans’ awareness on certain issues, and determining which social responsibility projects resonate the most by analyzing fans’ social media interactions. Analyzing fan behavior on digital platforms can help sports clubs make their social responsibility strategies more effective. Future studies can support these analyses with field studies and provide more comprehensive evaluations.

## 7 Conclusions and limitations

The social media posts regarding social responsibility show that the approaches of fan groups to the digital age are much more innovative. On the other hand, the content and distribution of the posts also reveal that the clubs are trapped in their own cultural areas, their own echo chambers. Despite this, especially Çarşı and UNIBJK groups have shown their leadership in addition to their inclusion in this field with creative and innovative social responsibility projects. The fact that every fan group within the scope of the study participates in social responsibility projects is important in terms of showing that awareness has been created among the fan groups on this issue.

When looked at in general, it should be said that a process of social responsibility is being carried out through football and connected to the Agenda Setting Approach. In addition, it is seen that social responsibility is increasingly becoming a strategic necessity among sports stakeholders and is also used as a means of reinforcing legitimacy.

The study is limited in that it covers 150 Instagram posts dating back to April 12, 2021. The study can be expanded by addressing the posts in different ways in terms of duration, number, and comments. The study, which is limited in that it addresses certain fan groups in Turkey, can be expanded to include different fan groups and different stakeholders. The study, which examines social responsibility projects in social media posts, can be designed more comprehensively by combining it with financial and administrative information that can be obtained about the projects. Although the fan groups of major football teams carry out significant social responsibility activities on social media platforms, evaluating these activities through social media may bring with it some biases. Representation, visibility and confirmation biases indicate that analyses based on social media posts may be limited. Future research should support social media data with field studies and address the actual social responsibility participation levels of football fans more comprehensively.

## Data Availability

The raw data supporting the conclusions of this article will be made available by the authors, without undue reservation.
